# Lacerations of the Female Perineum and Vesico-Vaginal Fistula

**Published:** 1874-02

**Authors:** 


*Lacerations of the Female Perineum and Vesico-Vaginal Fistula: Their History and Treatment.* By D. Hayes Agnew, M. D., etc., with numerous illustrations. Philadelphia: Lindsay & Blakiston, 1873. Buffalo: Theo. Butler & Son.

These papers have already been published, the first, in the *Pennsylvania Hospital Reports*, the second in the *Philadelphia Medical and Surgical Reporter*. The History of Lacerated perineum is given to some extent, and the different methods which have been pursued to prevent the occurrence of the accident or to remedy it when it has taken place, are described in detail. The author prefers the metallic suture secured by perforated shot, to the quilled suture. He gives a description of his operation, and relates the history of several cases. The conclusions that he arrives at are,

1st. That laceration of the perineum and the recto-vaginal septum can be satisfactorily cured at a single operation.

2d. That by the peculiar method of inserting the first suture (introduced three-fourths of an inch from the margin and below the lowest point of the wound, carried through the middle of the septum just above the line of denudation and carried through the corresponding part of the other side,) there is no necessity for a series of stitches to close the septum, independent of those used for the closure of the perineum.

3d. That the interrupted can be substituted for the quill suture.

4th. That the division of the sphincter is not necessary to a cure.

5th. That the superficial sutures may be dispensed with.

The article concludes with a resumé of the literature of the subject.

The article upon Vesico-Vaginal Fistula is the most extended of the two, and is a very able review of the subject. As in the first paper the history of the subject is given, its causes and the various methods of relief are dwelt upon to some extent. After a description of his own mode of operation, which does not differ materially from the methods usually pursued, the history of eighteen cases are given, in which Dr. Agnew operated for fistula. They all contain points of more or less interest, and make a valuable addition to the statistics of the operation. The papers are well written and give a clear and concise statement of the methods to be pursued in the operation for lacerated perineum and vesico-vaginal fistula, and are a valuable contribution to practical surgery.

